# Genome Sequence of *Penicillium capsulatum* Strain ATCC 48735, a Rare *Penicillium* Species Used in Paper Manufactories but That Recently Caused Invasive Infection

**DOI:** 10.1128/genomeA.00307-15

**Published:** 2015-05-21

**Authors:** Ying Yang, Min Chen, Zongwei Li, Abdullah M. S. Al-Hatmi, Qiang Ye, Huipeng Chen, Shengqi Wang, Wanqing Liao, Junzhi Wang

**Affiliations:** a Beijing Institute of Biotechnology, Beijing, China; b Department of Dermatology, Shanghai Key Laboratory of Molecular Medical Mycology, Changzheng Hospital, Shanghai, China; c CBS-KNAW Fungal Biodiversity Centre, Utrecht, the Netherlands; d Institute of Biodiversity and Ecosystem Dynamics, University of Amsterdam, Amsterdam, the Netherlands; e Beijing Institute of Radiation Medicine, Beijing, China; f Directorate General of Health Services, Ibri Hospital, Ministry of Health, Ibri, Oman; g National Institutes for Food and Drug Control, Beijing, China

## Abstract

The genus *Penicillium* phylogenetically belongs to *Trichocomaceae*, with approximately 300 reported species. The majority of these species are saprobic and commonly occur in soil. This paper reports the genome sequence of *Penicillium capsulatum* strain ATCC 48735, a rare *Penicillium* species used in paper manufactories and that was recently reported as a human-invasive opportunist.

## GENOME ANNOUNCEMENT

*Penicillium capsulatum* is well known to produce extracellular enzymes, which are mainly used in paper production, but they recently were reported to cause human infections in type 2 diabetes patients in China (1–3). The prevalence of *P. capsulatum* in nature is high and extensively used in the paper industry (1, 2); however, routine molecular analysis is not likely sufficient to reveal the hidden genetic basis of the role of *P. capsulatum*, especially the transition from being a production microorganism in paper manufactories to an emerging fungal pathogen (4, 5).

There are no whole-genome data of *P. capsulatum* to date; therefore, we performed genome sequencing of *P. capsulatum* strain ATCC 48735 using a combined next-generation sequencing (NGS) platform based on the Illumina HiSeq 2500 and Pacific Biosciences RS II sequencing techniques. The project is part of a comparative genomics study designed to explore more understanding of the pathogenic potential in *P. capsulatum* as a production *Penicillium* species in paper manufactories.

The genomic DNA was extracted from *P. capsulatum* strain ATCC 48735, an environmental isolate originally collected from an exposed canvas in Gilbert Island, Republic of Kiribati. The strain was deposited and available at the American Type Culture Collection (ATCC). The genome of *P. capsulatum* strain ATCC 48735 was successively sequenced by the Pacific Biosciences RS II and Illumina HiSeq 2500 platforms. Approximately 211-fold sequence coverage was generated from a total of 4.4 Gb and 3.0 Gb raw data using PacBio RSII single-molecular real-time (SMRT) and Illumina platforms, respectively. The read data from the Illumina platform were used to correct the assembly sequence based on the PacBio platform, leading to 48 single-nucleotide polymorphisms (SNPs) and 29 indels. Furthermore, the results from CEGMA showed that 97.18% (241/248) of the core eukaryotic genes mapped against the assembly genome of strain ATCC 48735, which also indicated that our genome assembly has a high coverage of the coding region. The assembled genome size is approximately 34.37 Mb, with a contig *N*_50_ of 3,189 kb and a G+C content of 49.07%. InterProScan was used to determine the functional annotations, motifs, and domains of all prediction genes, resulting in a total of 11,076 gene models with 1,807.7 bp average coding sequence length and 174 tRNAs. Meanwhile, approximately 1.42 Mb of the repeated regions was found in the genome of strain ATCC 48735, which accounts for 4.22% of the genome size. Among them, the interspersed repeat was the majority predominant type (85.1%).

### Nucleotide sequence accession numbers.

This whole-genome shotgun project has been deposited at DDBJ/EMBL/GenBank under the accession no. JPLQ00000000. The version described in this paper is version JPLQ01000000.
